# Up-regulation of IRF-3 expression through GATA-1 acetylation by histone deacetylase inhibitor in lung adenocarcinoma A549 cells

**DOI:** 10.18632/oncotarget.18371

**Published:** 2017-06-06

**Authors:** Lu-Lu Wang, Lan-Bo Zhou, Jin Shu, Nan-Nan Li, Hui-Wen Zhang, Rui Jin, Li-Li Zhuang, Guo-Ping Zhou

**Affiliations:** ^1^ Department of Pediatrics, The First Affiliated Hospital, Nanjing Medical University, Nanjing, Jiangsu Province, China; ^2^ Grade 2013 Clinical Class 7, The First School of Clinical Medicine, Nanjing Medical University, Nanjing, Jiangsu Province, China; ^3^ Department of Pediatric Respiration, Affiliated Wuxi People's Hospital, Nanjing Medical University, Wuxi, Jiangsu, China

**Keywords:** interferon regulatroy factor 3, GATA-1, acetylation, histone deacetylase inhibitor, lung adenocarcinoma

## Abstract

Interferon regulatory factor 3 (IRF-3) is an important transcription factor for interferon genes. Although its functional activation by viral infection has been widely explicated, the regulatory mechanism of IRF-3 gene expression in cancer cells is poorly understood. In this study, we demonstrated treatment of lung adenocarcinoma A549 cells with trichostatin A (TSA) and valproic acid (VPA), two different classes of histone deacetylase inhibitors, strongly stimulated IRF-3 gene expression. Truncated and mutated IRF-3 promoter indicated that a specific GATA-1 element was responsible for TSA-induced activation of IRF-3 promoter. Chromatin immunoprecipitation and electrophoretic mobility shift assay showed that TSA treatment increased the binding affinity of GATA-1 to IRF-3 promoter. Using immunoprecipitation assay and immunoblotting, we demonstrated that TSA increased the level of acetylated GATA-1 in A549 cells. In summary, our study implied that TSA enhanced IRF-3 gene expression through increased GATA-1 recruitment to IRF-3 promoter and the acetylation level of GATA-1 in lung adenocarcinoma A549 cells.

## INTRODUCTION

IRF-3 is well known in virus and double-stranded RNA-mediated induction of a large set of cellular antiviral proteins, acting as an important mediator in interferon immunosystem. Previous studies showed transcription factors bound to mouse and human IRF-3 promoters, dramatically regulating their transactivations [1–3]. Recently, studies uncovered IRF-3 emerged as a key tumor suppressor in DNA damage response and cancer cell growth inhibition. Ectopic expression of IRF-3 decreased melanoma and cervical cancer cells growth rate *in vivo* and *in vitro*, while suppressed IRF-3 block B-lymphoid cell differentiation in acute lymphoblastic leukemia [4–6]. IRF-3 was also involved in lung cancer that decreased and mutated IRF-3 gene expressions were found in non-small-cell lung cancer (NSCLC) patients, altering the immunoregulatory function in tumorigenesis [7, 8]. However, by which IRF-3 was activated in NSCLC cells have not been well documented yet.

Gene transcriptional activity can be modulated by alternation of DNA binding properties of key histones and transcription factors. Histone deacetylase inhibitor (HDACi) can specifically accumulate acetylated histones and non-histone proteins to certain tumor genes, playing a great role in cell proliferation, differentiation, cell-cycle arrest and tumor metastasis. The molecular mechanism of HDACi in lung cancer cells was currently under investigation. Hichino et al. demonstrated that A549 cells proliferation was decreased by TSA, which was partially rescued by ectopic expression of claudin-2 [9]. Entinostat, as a kind of HDACi drugs, has been investigated in SALL4 positive lung caners [10]. HDACi also inhibited the hypoxia-induced cisplatin resistance and mutant p53-induced instability in NSCLC cell lines [11]. These results, together with possible cross-talk between lung adenocarcinoma (LADC) and IRF-3, suggest that IRF-3 may be the target of HDACi in regulating the tumorigenesis in LADC cells.

In this study, we demonstrated novel findings that TSA increased the gene expression of IRF-3 by enhancing GATA-1 binding affinity to a specific binding site (nt -85) in IRF-3 promoter. Mutation of GATA-1 element of IRF-3 promoter destroyed TSA-induced IRF-3 transactivation in A549 cells. Furthermore, TSA increased the acetylated GATA-1 level in A549 cells which was partially attributed to p300 colocalization. In summary, these data indicate TSA activates IRF-3 transcriptional activity via an acetylation dependent manner in lung adenocarcinoma A549 cells.

## RESULTS

### HDACi treatment results in an increase of IRF-3 expression

**Figure 1:**
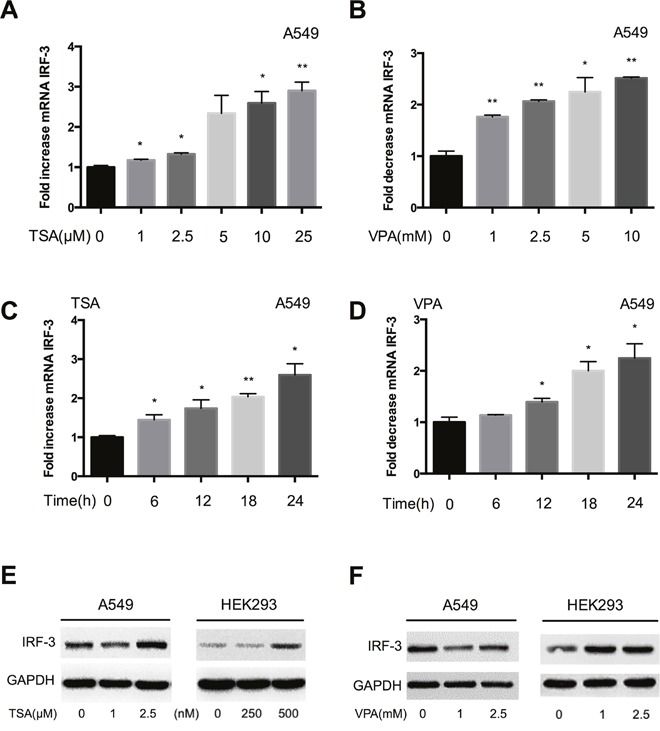
Up-regulation of IRF-3 gene expression in HDACi treated cells **(A)** The IRF-3 mRNA expression was examined in A549 cells after different dose of TSA or **(B)** VPA treatment for 24 h. **(C)** TSA or **(D)** VPA stimulated IRF-3 mRNA expression in a time-dependent manner. Bar represents the mean ± S.D. from three independent experiments (*p<0.05; **p<0.01). **(E)** TSA or **(F)** VPA induced IRF-3 protein increase was determined by immunoblotting in A549 and HEK293 cells. GAPDH was shown as a loading control.

In this study, we first examined the effect of HDACi on IRF-3 gene expression. The result showed HDACi improved IRF-3 mRNA in a time- and dose-dependent manner in A549 and HEK293 cells. There was a 2-3 fold increase of IRF-3 mRNA in cells treated with 25 μM TSA and 10 mM VPA (Figure 1A, 1B, and Supplementary Figure 1). Maximum IRF-3 mRNA expression induced by 2.5 μM TSA and 5 mM VPA was observed at 24 h in A549 cells (Figure 1C and 1D). Immunoblotting also confirmed IRF-3 protein level was elevated by HDACi in a dose-dependent manner (Figure 1E and 1F).

### Activation of the IRF-3 promoter by TSA requires a specific GATA-1 element

**Figure 2:**
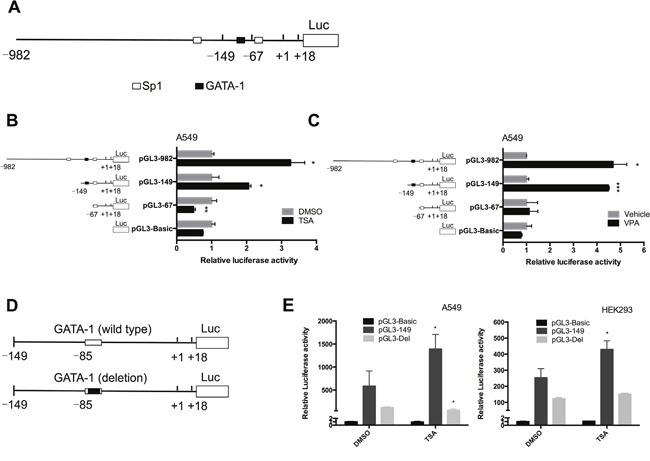
GATA-1 element is responsible for the active response of IRF-3 to TSA **(A)** Mapping of the IRF-3 promoter showed the transcription factors which are subject to acetylation bound to nt -149 to +18 region of promoter. **(B and C)** The luciferase activity of truncated IRF-3 promoter plasmids was measured in TSA or VPA treated A549 cells. Results are expressed as a fold of the untreated control that is set as 1. **(D and E)** IRF-3 promoter deletion construction and luciferase activity measurement in TSA treated cells. Bar represents the mean ± S.D. from three independent experiments (*p<0.05; **p<0.01; ***p<0.001).

We used the Matlnspector (http://www.genomatix.de//matinspector.html) to inspect of the promoter 5’-flanking region of human IRF-3 gene. As shown in Figure 2A, several putative elements subject to acetylation were found: Sp1 elements at nt -250 and nt -64, and GATA-1 element at nt -85. To further elucidate specific promoter region that is responsible for HDACi-induced activation of the IRF-3 promoter, a series of 5’-flanking region of IRF-3 gene were inserted to pGL3-Basic as before [2]. As shown in Figure 2B and 2C, in response to 2.5 μM TSA or 5 mM VPA, the transcriptional activity of pGL3-982 and pGL3-149 was increased to a 2-5 fold elevation compared to pGL3-67. This result indicated that the region from nt -149 to -67 containing GATA-1 element (nt -85 to -73) could markedly response to TSA treatment. Thus, we made site deletion of GATA-1 element (GGTCGATAACCGG→GGTGG) from the wild plasmid pGL3-149 to define the role of this region (Figure 2D). As shown in Figure 2E, TSA-induced IRF-3 transcriptional activation was eliminated when the GATA-1 binding site was deleted, suggesting that GATA-1 element was responsible for TSA-induced activation of IRF-3 promoter.

### TSA enhanced GATA-1 binding affinity to IRF-3 promoter *in vivo* and *in vitro*

**Figure 3:**
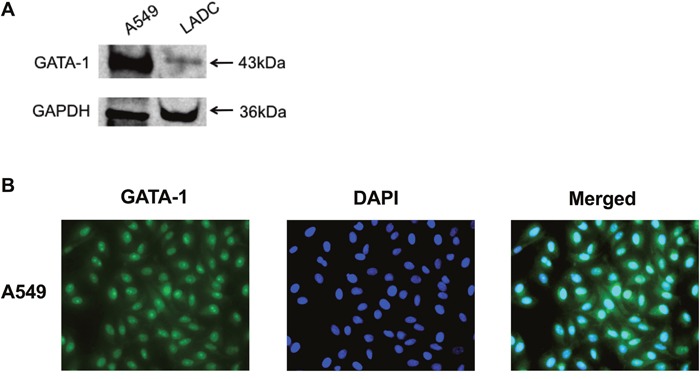
GATA-1 expression in human LADC tissue and A549 cells **(A)** GATA-1 protein expression was analyzed by immunoblotting in LADC tissue and A549 cells. **(B)** A549 cells were cultured for 24 h and analyzed by immunofluorescence. GATA-1 was immunostained in green and nuclei was stained with DAPI (blue).

**Figure 4:**
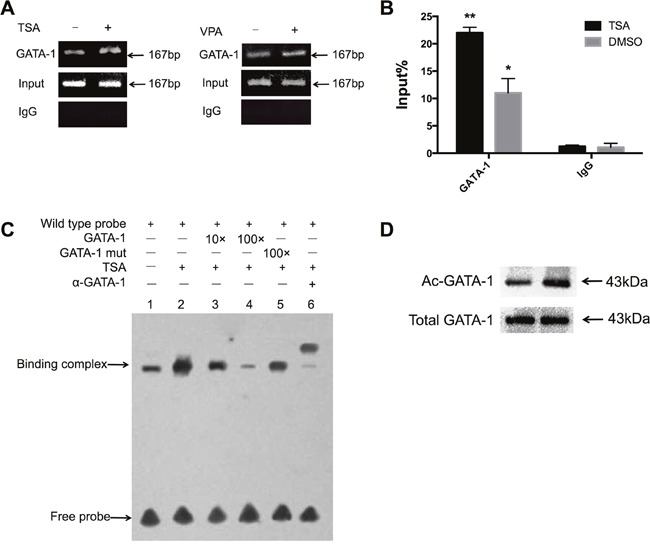
TSA increases the binding affinity of GATA-1 to IRF-3 promoter **(A)** A549 cells were treated with or without TSA and VPA for 24 h. Input indicated 10% total chromatin DNA subjected for RT-PCR analysis while IgG acted as a negative control. PCR products were 167 bp and were visualized by agarose gel electrophoresis and ethidium bromide staining. **(B)** ChIP-qPCR demonstrated significant accumulation of GATA-1 in the IRF-3 promoter region. Bar represents the mean ± S.D. from three independent experiments (*p<0.05; **p<0.01). **(C)** A549 cells were treated with or without TSA for 24 h. EMSA was performed with a DNA probe harboring the GATA-1 element (nt -85 to -73) on the IRF-3 promoter as described in Materials and Methods. **(D)** Nuclear extracts from A549 cells were examined by immunoblotting using anti-Ac-lysine antibody. The amounts of total GATA-1 protein expressed in nuclei were also detected by immunoblotting.

The aberrant expression or DNA modification of GATA-2, GATA-4, GATA-5, and GATA-6 was found in lung cancer, but GATA-1 gene expression is seldom reported in LADC [12–14]. To explore the expression of GATA-1 in lung cancer, we detected GATA-1 protein expression in human LADC tissue and A549 cells (Figure 3A). Immunofluorescence assay further confirmed that GATA1 protein was mainly expressed in the nuclei of A549 cells (Figure 3B). To investigate *in vivo* how GATA-1 mediates the activation of the IRF-3 promoter by HDACi, we performed chromatin immunoprecipitation (ChIP) assay. The chromatin of A549 cells were immunoprecipitated with ani-GATA-1 and anti-IgG antibodies. Then, precipitated DNA was amplified by RT-PCR with primers flanking the IRF-3 promoter region (nt -149 to +18). The results showed treatment with TSA or VPA significantly facilitated the binding affinity of GATA-1 to this region (Figure 4A). ChIP-qPCR assay demonstrated the significant recruitment of GATA-1 to the IRF-3 promoter region induced by TSA (Figure 4B).

To delineate whether TSA improves GATA-1 binding affinity to this putative binding site *in vitro*, we performed electrophoretic mobility shift analysis (EMSA) with A549 cells nuclear extracts. As shown in Figure 4C, TSA treatment caused an obvious increase of binding complex (lane 2, Figure 4C) compared to that without TSA treatment (lane 1, Figure 4C). The binding complex was significantly reduced by 10- and 100- fold unlabeled probes competition, but mutated probe could not eliminate it (lane 3, 4, and 5, Figure 4C). Supershift study showed the binding complex was blocked when incubated with anti-GATA-1 antibody (lane 6, Figure 4C). Taken together, these results demonstrated that TSA enhanced GATA-1 binding affinity to IRF-3 promoter *in vivo* and *in vitro*.

Immunoprecipitation assay and immunoblotting were done to further explore whether TSA-induced activation of IRF-3 promoter is attributed to acetylation level of GATA-1 in A549 cells. The result showed acetylated GATA-1 was elevated in response to TSA, and immunoblotting analysis revealed no difference in total GATA-1 protein expression between TSA treated and untreated A549 cells, suggestive of a conformational change in GATA-1 (Figure 4D).

In this study, a putative Sp1 binding site at nt -64 was close to the GATA-1 element in IRF-3 promoter (Figure 2A). Previously, Sp1 was reported to stimulate the promoter activity of some genes [3, 15]. Thus, to identify if Sp1 binding affinity to IRF-3 promoter could be stimulated by TSA, we carried out ChIP assay. As shown in Supplementary Figure 2, Sp1 bound to nt -149 to +18 of IRF-3 promoter in A549 cells but had no response to TSA treatment. In conclusion, these results suggested that GATA-1 but not Sp1 could enhance the IRF-3 promoter activity induced by TSA in A549 cells.

### p300 was involved in TSA-induced GATA-1 acetylation

**Figure 5:**
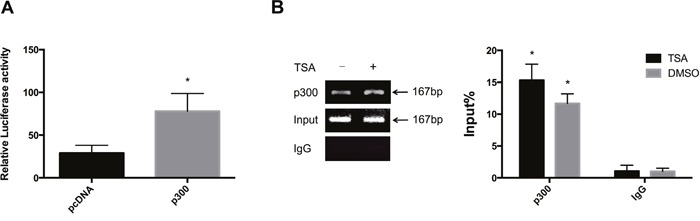
Interaction of p300 in TSA-induced GATA-1 acetylation **(A)** A549 cells were cotransfected with pcDNA-p300 expression plasmid and pGL3-149 for luciferase assay. **(B)** ChIP assays showed the occupancy of p300 protein on nt -149 to +18 of IRF-3 promoter. Enrichment of p300 at this region relative to Input was quantitated. Bar represents the mean ± S.D. from three independent experiments (*p<0.05).

p300 was reported to acetylate GATA-1 within its dual-zinc finger domain and stimulate GATA-1 dependent transcription both *in vivo* and *in vitro* [16, 17]. Therefore, we examined the binding affinity of p300 to IRF-3 promoter to investigate the interaction between GATA-1 and p300. The plasmids pcDNA or pcDNA-p300 were co-transfected with pGL3-149 into A549 cells. Luciferase reporter assay showed p300 strongly increased the activity of pGL3-149 compared to pcDNA control (Figure 5A). Moreover, ChIP-qPCR assay demonstrated that *in vivo* occupancy of p300 at nt -149 to +18 was elevated by TSA (Figure 5B). Thus, we speculated that TSA recruited p300 to the GATA-1 element in IRF-3 promoter and enhanced the binding affinity of GATA-1.

## DISCUSSION

In the field of lung cancer, anti-tumor responses have been observed in the clinical research by treating NSCLC patients with HDACi alone or combined with chemotherapy, such as SAHA and CI-994 [18, 19]. Studies about IRF-3 revealed it is a tumor suppressor gene in cancer cells, directly or indirectly inhibiting cell growth [6] [20, 21]. Oshita et.al observed higher expression of IRF-3 in tumors of surviving patients with stage I NSCLC than that in patients who succumbed from cancer [22]. Thus, it is meaningful to investigate how IRF-3 is regulated in lung cancer. In this study, we selected two different kinds of HDACi to do research in lung adenocarcinoma A549 cells, TSA from hydroxamic acids and VPA from carboxylic acids. Our results demonstrated the significant up-regulation of IRF-3 gene expression by HDACi and identified that a specific GATA-1 element was the major TSA response domain responsible for IRF-3 transactional activation.

GATA-1, the founding member of the GATA factor family contains acetylation sites located predominantly within its C-terminal tails of dual-zinc finger domain [23]. Prior reports demonstrated acetylated and phosphorylated GATA-1 influenced gene expression in tumorigenesis, such as breast cancer and hematologic malignancies [24–26], but there is no study about GATA-1 in LDAC. Here, we demonstrated in A549 cells that TSA enhanced GATA-1 recruitment to IRF-3 promoter and facilitated the acetylation level of GATA-1. The same pattern was found in other genes, such as hWNK4 and Polo-like kinase 2 promoters, of which TSA-induced transactivation relied on the specific GATA-1 binding sites [27, 28]. To our knowledge, it is the first study demonstrating the TSA-induced acetylated GATA-1 increased IRF-3 gene expression in A549 cells.

Except for HDACi, several cofactors are also responsible for the acetylation of GATA-1, such as histone acetyltransferase CBP/p300, FOG 1, HDAC5, TRAP, and Brd3 [16, 29–32]. Our study suggested TSA induced p300 colocalization to GATA-1 element in IRF-3 promoter. However, we do not know the direct relationship between p300 and GATA-1 in A549 cells. Wu et.al performed coimmunoprecipitation assays and overexpression experiment to determine the cooperation of these two factors facilitated by PML4 [33]. Thus, further investigation is required to elucidate the direct relationship of p300 and GATA-1 in A549 cells.

We observed a putative Sp1 binding site (nt -64) which is close to the GATA-1 element in IRF-3 promoter, while Xu et al. reported a different Sp1 binding site at nt -105 to -95 predicted by AliBaba2.1 software and exogenous Sp1 increased IRF-3 transcriptional activity through this region [3]. However, in our study, no significant difference was found of the Sp1 binding affinity to nt -149 to +18 in IRF-3 promoter with or without the treatment of TSA *in vivo* (Supplementary Figure 2), implying that transcription factor Sp1was not significant in TSA induced IRF-3 transactivation.

Taken together, we demonstrated novel findings that TSA stimulated IRF-3 gene expression by enhancing GATA-1 binding affinity to a specific element in IRF-3 promoter. Besides, we proposed p300 might be recruited to IRF-3 promoter and be responsible for GATA-1 acetylation in the TSA induced effect on IRF-3 transactivation. These observations suggest targeting IRF-3 by HDACi may be a novel approach in LADC therapy.

## MATERIALS AND METHODS

### Cell culture and reagents

HEK293 and A549 cells (ATCC) were maintained in Dulbecco's modified Eagle's medium with 10% fetal bovine serum, penicillin and streptomycin. Cells were cultured in a humidified atmosphere of 5% CO_2_ maintained at 37 °C. TSA and VPA were purchased from Sigma-Aldrich (USA). TSA was dissolved in dimethyl sulfoxide (DMSO) and VPA was dissolved in distilled water.

### Human LADC samples

LADC tissue and adjacent normal tissue were procured after surgical resection from patients with LADC and stored at -70 °C in the First Affiliated Hospital of Nanjing Medical University. This study protocol was approved by the the Ethics Committees of the First Affiliated Hospital of Nanjing Medical Universtiy(#1102/2008). All samples were collected after the informed consents of patients were written.

### Plasmids

Transcriptional start site of human IRF-3 promoter was set as +1. The truncated human IRF-3 promoter plasmids pGL3-982, pGL3-149, and pGL3-67 were constructed as before [9]. The software Matlnspector (http://www.genomatix.de//matinspector.html) were used to search for IRF-3 promoter sequence for potential transcription factors target sites which are subject to acetylation. Deletion of pGL3-149 (pGL3-Del) was generated by PCR using the Site-Directed Mutagenesis Kit (Takara). Primer sequence for mutation were synthesized as 5’-GGCCCAGCGTAGAAAGGGCGGAACGCT-3’ (sense); 5’-ACCCGGCCCAGTGCGCAGGCGCG-3’ (anti-sense). All constructs were confirmed by sequencing without coding frame shifts. The plasmid pcDNA-p300 for overexpression experiment was a kind donate from Dr. Tony Kouzarides (The Gurdon Institute, UK) and the corresponding control plasmid pcNDA3.1/Myc-HisA was purchased from Invitrogen (Carlsbad, CA, USA).

### Cell transfection and luciferase assays

A total of 1.5×10^4^cells/well cells were seeded into 96-well plates for 24 h followed by truncated IRF-3 promoter plasmids (100 ng) and pRL-TK (4 ng) plasmid transfected into cells using Lipofectamine^™^ 3000 (Invitrogen). After 24 h of transfection, the cells were incubated with or without HDACi for another 24 h before analysis of luciferase assays. For p300 overexpression experiment, pcDNA3.1 or pcDNA-p300 were transfected with pGL3-149 into cells for 24 h followed by the promoter activity detection. Promoter activity was detected by a Dual-luciferase Reporter Assay System (Promega) and was normalized to the activity of pRL-TK. Results were representative of at least three independent experiments performed in triplicate.

### Reverse transcription and quantitative real-time PCR

Total RNA was extracted from cells and sbusequently reverse transcribed to cDNA with equal RNA volume (1μg) using the PrimeScript RT Master Mix Perfect Real Time kit (Takara). Real-time PCR was performed on the Applied Biosystems Step One Plus Real-Time PCR System. Primer sequences for detection of IRF-3 mRNA expression were: 5’-GGACCCTCACGACCCACATA-3’ (sense) and 5’- CCATGTTACCCAGTAACTCATCCAG-3’(anti-sense); the primer sequences for detection of GAPDH mRNA expression were: 5’-TGGTATCGTGGAAGGACTCATGAC-3’ (sense) and 5’- TGCCAGTGAGCTTCCCGTTCAGC-3’ (anti-sense). All the reactions were repeated at least three times. Gene expression levels were normalized to GAPDH and relative expression level was calculated with the comparative CT method.

### Immunoblotting

Cells were treated with TSA or VPA at different concentration for 48 h. Total protein was extracted from cells and LADC tissue with lysis buffer containing protease inhibitor cocktail (Keygentec, China) and the concernrations were determined by the BCA Protein Assay Kit (Beyotime). Equal amount of protein were separated by sodiumdodecyl sulfate-polyacrylamide gel electrophoresis (SDS-PAGE) and transferred to a nitrocellulose membrane (Millipore). The membrane were blocked with 5% nonfat milk in TBST (0.1% Tween-20) and then incubated with anti-IRF-3 antibody (Abcam), anti-GATA-1 antibody (Abcam), or anti-GAPDH antibody (Bioworld) at 4 °C overnight at a dilution of 1:2000-1:4000, followed by anti-rabbit IgG (Cell signaling) or anti-mouse IgG (Jackson ImmunoResearch Laboratories) at a dilution of 1:5000. Intensity of bands was measured using an ECL imager.

### Immunofluorescence

A549 cells were seeded on coverslips, grown to 50% confluence, and fixed in paraformaldehyde at room temperature for 60 min. The cells were blocked with 5% bovine serum albumin (BSA) and incubated with the anti-GATA-1 overnight at 4°C and subsequently with FITC-labeled secondary antibody (Jackson ImmunoResearch Laboratories) after washed three times in PBS and 0.4% Triton x-100. Nuclei were stained with DAPI (Beyotime, China) before mounting onto slides. Confocal scanning analysis was performed by using a Nikon microscope in accordance with established methods.

### ChIP-qPCR

ChIP assays were performed with the EZ-Magna ChipTM A kit (Millipore) using the manufacturer's instructions. A549 cells were treated with 2.5 μM TSA or 5 mM VPA for 24 h and cross-linked with 1% formaldehyde at room temperature for 10 min. The chromatin of A549 cells was sonicated on ice and then immunoprecipitated with anti-IgG antibody (Millipore), anti-GATA-1 antibody (Abcam), anti-Sp1 antibody (Santa Cruz), and anti-p300 antibody (Santa Cruz). After reverse cross-linking and DNA purification, DNA from input (1:10 diluted) or immunoprecipitated samples were assayed by RT-PCR. The 1% input and immunoprecipitated DNA were quantitatively compared by real time PCR using SYBR Green (TaKaRa). The IRF-3 promoter region, nt -149 to +18, containing GATA-1 element was amplified. Primer pairs for this region were: 5’-GTCTCCTCCACTGAACTCGTAC-3’ (sense) and 5’-GCCCTTTTTTGGGTTTCC-3’ (anti-sense).

### EMSA

Two double-stranded oligonucleotides were synthesized (Invitrogen): wild-type 5’-biotinylated double-stranded oligonucleotides containing the GATA-1 binding motif 5’-GGCCGGGTCGATAACCGGCCCA-3’ and the mutant oligonucleotide with underlined mutated bases: 5’-GGCCGGGTCATGCACCGGCCCA-3’. Detection of the GATA-1 oligonucleotide complex was performed using the Lightshift Chemiluminescent EMSA Kit (Thermo Scientific). After 24 h TSA treatment, nuclear extracts of A549 cells were prepared and incubated in a buffer containing 10 mM Tris, 50 mM KCl, 1 mM DTT (pH 7.5), 7.5% glycerol, and 75 ng/ml poly (dI-dC) for 20 min at room temperature. After incubation, 1 μl of the biotinylated oligonucleotides and 1 μl anti-GATA-1 antibody were incubated at room temperature for 40 min. The DNA-protein complexes were resolved on a 6% polyacrylamide gel in 0.5% TBE buffer for 1 h. After the DNA was cross-linked to the membrane, ECL system was performed to measure the band.

### Immunoprecipitation

Nuclear extracts from A549 cells with or without TSA treatment were prepared using NE-PER (Thermo Scientific). According to the manufacturer's protocol, protein A/G-agarose magnetic beads (Thermo Scientific) were incubated with 5 μg anti-GATA-1 antibody at room temperature for 15 min. After wash, antibody-crosslinked beads was incubated with cell lysate at room temperature for 1 h. The immunoprecipitates were subjected to SDS-PAGE followed by immunoblotting using an anti-Ac-lysine antibody (Santa Cruz).

## SUPPLEMENTARY MATERIALS AND FIGURES


