# Supplementary material for: Up-regulation of IRF-3 expression through GATA-1 acetylation by histone deacetylase inhibitor in lung adenocarcinoma A549 cells
Source: Oncotarget. 2017 Jun 6;8(44):75943–51. doi: 10.18632/oncotarget.18371 (PMC5652676; doi:10.18632/oncotarget.18371)
Supplement: Supplementary file 1 [file oncotarget-08-75943-s001.pdf]

Up-regulation of IRF-3 expression through GATA-1 acetylation by histone deacetylase inhibitor in lung adenocarcinoma A549 cells

SUPPLEMENTARY FIGURES

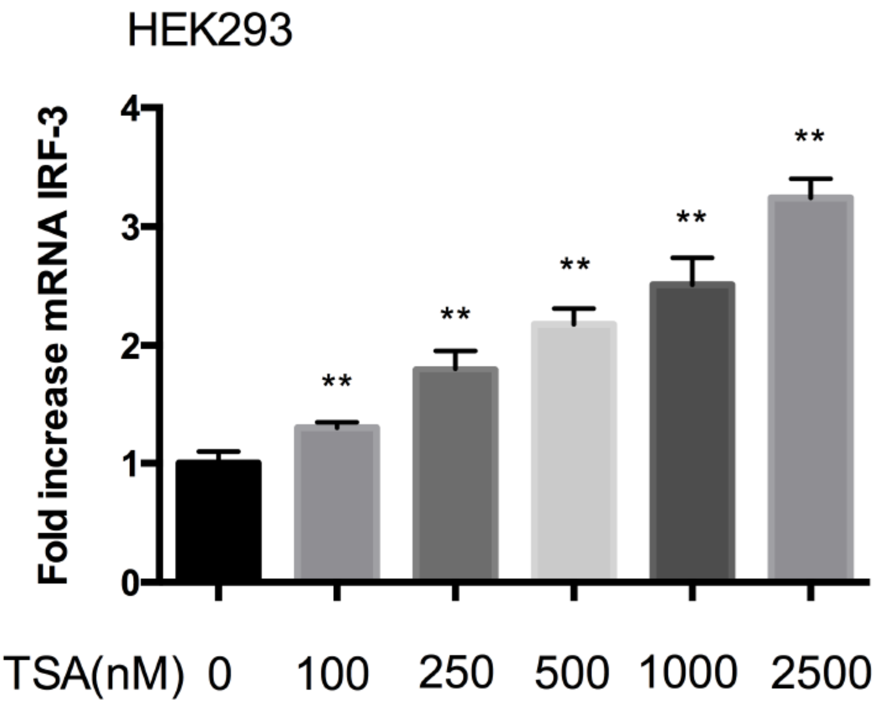

Supplementary Figure 1: IRF-3 mRNA expression in TSA treated HEK293 cells. The IRF-3 mRNA expression was examined in HEK293 cells after different dose of TSA treatment for 24 h. Bar represents the mean ± S.D. from three independent experiments (***p*<0.01).

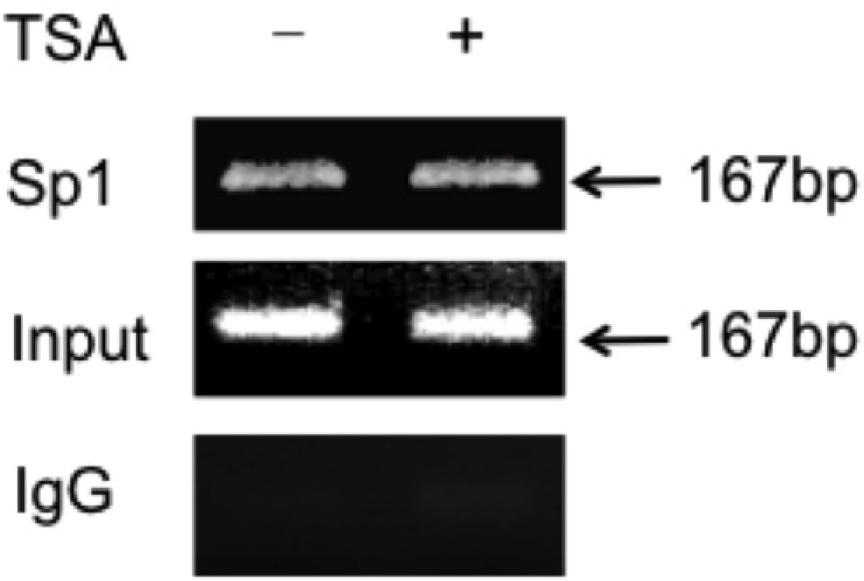

Supplementary Figure 2: ChIP assays showed Sp1 binding affinity to IRF-3 promoter (nt -149 to +18) with or without the TSA treatment. PCR products were detected by agarose gel electrophoresis.
